# 3D Ultralight Hollow NiCo Compound@MXene Composites for Tunable and High-Efficient Microwave Absorption

**DOI:** 10.1007/s40820-021-00727-y

**Published:** 2021-10-11

**Authors:** Hui-Ya Wang, Xiao-Bo Sun, Shu-Hao Yang, Pei-Yan Zhao, Xiao-Juan Zhang, Guang-Sheng Wang, Yi Huang

**Affiliations:** 1 School of Chemistry, Beihang University, Beijing, 100191 People’s Republic of China; 2 College of Chemistry and Materials Engineering, Beijing Technology and Business University, Beijing, 100048 People’s Republic of China; 3 School of Materials Science and Engineering, Nankai University, Tianjin, 300350 People’s Republic of China

**Keywords:** 3D hollow hierarchical architecture, Tunable EAB, High-performance microwave absorption, Ultralight

## Abstract

The 3D hollow hierarchical architectures tend to be designed for inhibiting stack of MXene flakes to obtain satisfactory lightweight, high-efficient and broadband absorbers. Herein, the hollow NiCo compound@MXene networks were prepared by etching the ZIF 67 template and subsequently anchoring the Ti_3_C_2_T_*x*_ nanosheets through electrostatic self-assembly. The electromagnetic parameters and microwave absorption property can be distinctly or slightly regulated by adjusting the filler loading and decoration of Ti_3_C_2_T_*x*_ nanoflakes. Based on the synergistic effects of multi-components and special well-constructed structure, NiCo layered double hydroxides@Ti_3_C_2_T_*x*_ (LDHT-9) absorber remarkably achieves unexpected effective absorption bandwidth (EAB) of 6.72 GHz with a thickness of 2.10 mm, covering the entire Ku-band. After calcination, transition metal oxide@Ti_3_C_2_T_*x*_ (TMOT-21) absorber near the percolation threshold possesses minimum reflection loss (RL_min_) value of − 67.22 dB at 1.70 mm within a filler loading of only 5 wt%. This work enlightens a simple strategy for constructing MXene-based composites to achieve high-efficient microwave absorbents with lightweight and tunable EAB.

**Supplementary Information:**

The online version contains supplementary material available at 10.1007/s40820-021-00727-y.

## Highlights

- Ultralight 3D NiCo compound@MXene nanocomposites that inherited hollow polyhedral skeleton and excellent conductive network were fabricated.
- Excellent electromagnetic absorption performance was achieved with optimal RLmin value of − 67.22 dB and ultra-wide EAB of 6.72 GHz under the low filler loading.
- Electromagnetic parameters and microwave absorption property can be distinctly or slightly regulated by adjusting the filler loading and decoration of Ti_3_C_2_T_x_ nanoflakes.

**Supplementary Information:**

The online version contains supplementary material available at 10.1007/s40820-021-00727-y.

## Introduction

Currently, the burgeoning 5G telecommunication is a new-generation technology with faster transmission speed, larger network capacity and shorter delay. However, it cannot be ignored the growing hazard of the ensuing electromagnetic interference (EMI) and electromagnetic (EM) radiation, which directly affects human physical health and normal operation of electronic devices. Hence, the intense demands for suppression of harm EM pollution/radiation accelerate the research on high-performance microwave absorption (MA) materials in the GHz range [1–3].

Over the past few decades, considerable efforts have been focused on selecting the proper components and structures. Generally, two-dimensional (2D) materials such as graphene [4], g-C_3_N_4_ [5], and MXene [6] exhibit more in-plane reflection and multiple reflections between the layers as well as superior in-plane electron transfer, benefiting to promote the attenuation of EM wave [7]. Lately, transition metal carbides and nitrides (MXene), as a typical and novel 2D material, have garnered increasing attention and expansive application prospects in both EMI shielding and MA field owing to its extraordinary inherent conductivity, large specific surface area, superior mechanical properties and accordion-like morphology [8, 9]. Besides, rich surface functional group, interfaces and intrinsic defects would contribute to the increase in dipole and interface polarization which are beneficial to form dielectric loss, making MXene become a promising candidate for EM absorbing materials [10].

However, the 2D merits of MXene are still hindered by its self-restacking or aggregation on accounted of strong van der Waals interaction and hydrogen bond between adjacent nanosheets [11]. It is not conducive to the full manifestation of surface active sites, multiple reflection and scattering of electromagnetic wave, which further result in obvious decrease in MA ability. Introducing interlayer spacers, such as metal ions [12], metal oxide nanoparticles [13], polymers [14] or constructing 3D hollow architecture, has been proved to be effective strategies to resolve this issue [15, 16]. Li et al. [17] introduced Ni nanoparticles into multilayered gaps of the adjacent 2D MXene, which efficiently alleviated the self-restacking of MXene and displayed the distinct absorption property with reflection loss of − 50.5 dB. In addition, the 2D MXene nanosheets enable to be constructed into special 3D structures via mechanical shearing [18], template method [19], self-assembly [20], spray drying [7] and so on. Zhao et al. [19] demonstrated on processing poly(methyl methacrylate) (PMMA) spherical as sacrificial templates to construct 3D macroporous frameworks. The results indicated these 3D MXene films exhibited much improved performance compared to multilayer MXenes. Liang et al. [2] prepared Ni/MXene hybrids via co-solvothermal method, which displayed a strong reflection loss of − 52.6 dB and broad EAB of 3.7 GHz. Cui et al. [7] successfully fabricated 3D pleated RGO/MXene/Fe_3_O_4_ microspheres (FMCM) by ultrasonic spray technology. Benefiting from the 3D porous structure and reasonable composition, the FMCM composites showed excellent MA performance that the minimum reflection loss reached − 51.2 dB and EAB was 4.7 GHz at 2.9 mm. Although these structural engineering strategies are highly efficient, most of them are complicated with sophisticated post-processing or harsh preparation conditions, which easily induce oxidation and structural degradation [21]. Considering the device dependency and the integrity of MXene, it is an undoubtedly convenient and operable strategy to construct 3D hierarchical architecture by electrostatic self-assemble negatively charged MXene with suitable components.

Layered double hydroxides (LDHs), as a kind of lamellar material mixed with adjustable hydroxides containing divalent and trivalent metal ions, possess more abundant active sites than those of single metal compounds, which is helpful to achieve potentially valuable MA materials [22]. Furthermore, the LDHs tend to avoid skin effect derived from metal-based absorbents at high frequency and be favorable for the entry of EM waves due to their semiconductor characteristic. Compared with pure metal materials, the LDHs own better chemical stability to enlarge their practical applications [23]. Recently, metal organic frameworks (MOFs) are utilized as a promising self-template to construct LDH materials with hollow or core–shell structure, which normally equip with large surface areas and porous architecture to alleviate the stress and endow their enhanced MA performance [24, 25]. However, LDH monomers usually deliver limited local microcurrent networks, which directly result in poor conduction loss. Coupling with highly conductive materials is undoubtedly a feasible solution. For example, Zhao et al. [26] successfully synthesized CoNi@NCPs-rGO composites by pyrolyzing the ZIF-67@CoNi LDHs-GO precursor. The optimal RL_min_ of the compounds could reach up to − 58.2 dB and the EAB was 4.03 GHz at 2.5 mm with a filler loading of 30%. Wen et al. [23] prepared porous layered double hydroxide@carbon (LDH@C) composites through calcination and alkali treatment of Co_*x*_Al_*y*_-MOF-74. The existence of polarization loss, electrical loss and open void space contributed to substantially strong RL_min_ of − 45.7 dB and broaden EAB of 9.0 GHz at 3.7 mm (20 wt%). Wang et al. [27] constructed carbon fabric interconnected with NiFe LDH/MXene-derived FeNi_3_/TiO_2_ absorber, and adjust the content of FeNi_3_/TiO_2_ surrounding the percolation threshold to achieve superior absorption performance. Under the addition of 7.0 wt% NiFe LDH/MXene precursor, FeNi_3_/TiO_2_ displayed an optimal RL_min_ of − 58.0 dB and a broad EAB of 7.0 GHz at 2.5 mm. However, it is relatively rare that 3D hollow LDH nanocages derived from MOF combine with MXene to release self-restacking and further optimize EM wave absorption.

Herein, we designed a facile and controllable strategy to construct 3D hollow NiCo LDH@Ti_3_C_2_T_*x*_ MXene (LDHT) network with hierarchical structure by electrostatical self-assembly. The existence of 3D hollow NiCo LDH with low-conductivity could not only alleviate the skin effect efficiently, but also weaken the self-restacking of MXene nanosheets simultaneously. Moreover, large interflake cavity also dramatically promotes the impedance matching character and prolongs the propagation path of electromagnetic wave to enhance the dielectric loss. Based on the superiority of the unique structure and the synergy effects of multiple components, the LDHT absorbents with different contents of MXene exhibit outstanding MA capabilities. It is found that the RL_min_ values gradually shift to the lower frequency accompanied with reduction in EAB as increasing the decoration of Ti_3_C_2_T_*x*_ nanoflakes and filler loading in polyvinylidene fluoride (PVDF). In addition, regulating the mass of immobilized Ti_3_C_2_T_*x*_ flakes can subtly tune the absorbing property compared with adjusting filler content. For comparison, NiCo transition metal oxide (TMO)@Ti_3_C_2_T_*x*_ MXene (TMOT) with fine adjustment of adherent Ti_3_C_2_T_*x*_ MXene content were prepared by calcination to rationally manipulate the electromagnetic parameters as well as MA performance. This work highlights the optimized hollow framework structure of MOF-derived LDH anchored with MXene nanosheets and provides recent progress to construct MXene-based absorbers.

## Experimental Section

### Materials

Ti_3_AlC_2_ powders (400 mesh) were purchased from 11 Technology Co., Ltd (Jilin, China). Lithium fluoride (LiF), 2-Methylimidazole (C_4_H_6_N_2_), nickel nitrate hexahydrate (Ni(NO_3_)_2_·6H_2_O) and cobalt nitrate hexahydrate (Co(NO_3_)_2_·6H_2_O) were purchased from Macklin Co., Ltd (Shanghai, China). Hydrochloric acid (HCl, 37%), N,N-dimethylformamide (DMF), anhydrous ethanol (C_2_H_5_OH) and anhydrous methanol (CH_3_OH) wereobtained from Modern Oriental (Beijing) Technology Development Co., Ltd (Beijing China). All reagents were of analytical grade and used without further purification.

### Preparation of Ti_3_C_2_T_***x***_ MXene Nanosheets

Typically, 1.0 g of LiF was dispersed in HCl aqueous solution (9 M, 20 mL), followed by the slowly adding 1.0 g Ti_3_AlC_2_ within 5 min. After etching for 24 h at 35 °C under continuous stirring, the product was washed with deionized water and then centrifuged at 3500 rpm several times until the pH of 6. Afterward, the precipitate was ultrasonicated for 90 min under Ar flow and centrifugated for 1 h at 3500 rpm. Finally, a dark green Ti_3_C_2_T_*x*_ supernatant was collected.

### Preparation of Hollow NiCo LDH Nanocages

The ZIF 67 polyhedrons were synthesized according to the reported method [28]. Firstly, 0.32 g of as-prepared ZIF 67 precursor and 0.6 g of Ni(NO_3_)_2_‧6H_2_O were dispersed into 60 mL of ethanol, respectively. After mixing the two solutions, the reaction vessel was treated at 90 °C for 80 min. Subsequently, the hollow NiCo LDH nanocages can be collected by washing and centrifugation for several times with ethanol.

### Preparation of 3D Hollow NiCo LDH@Ti_3_C_2_T_***x***_ and NiCo TMO@Ti_3_C_2_T_***x***_ MXene Composites

The hierarchical NiCo LDH@Ti_3_C_2_T_*x*_ (LDHT) nanocomposites were prepared through an electrostatic self-assemble process. Initially, 0.03 g of NiCo LDH was uniformly dispersed in 20 mL of deionized water, and Ti_3_C_2_T_*x*_ nanosheets suspension (1.0 mg mL^−1^) were added. After stirring for 20 min, LDHT composites were obtained after freeze-drying, while NiCo TMO@Ti_3_C_2_T_*x*_ (TMOT) composites were fabricated by calcining LDHT composites at 350 °C under Ar atmosphere for 2 h with heating rate of 2 °C min^−1^. The resultant composites were denoted as LDHT-x and TMOT-x, where x (6, 9, 12 15, 18, 21, and 24) was the mass of Ti_3_C_2_T_*x*_ MXene suspension.

### Characterization

The crystal structure of the obtained products was identified by X-ray diffraction (XRD) pattern (Bruker D8 AVANCE X-ray diffractometer with Cu K*α*). The morphologies and nanostructures were observed with field emission scanning electron microscopy (SEM, JSM-7500F) and transmission electron microscopy (TEM, JEM-2100F). X-ray photoelectron spectroscopy (XPS) was characterized by Thermo Escalab 250Xi X-ray photoelectron spectrometer to analyze the surface chemical compositions. The zeta potential was conducted on Malvern Zetasizer Nano ZS (Malvern Instruments, USA). Nitrogen adsorption/desorption isotherms and pore structures of as-obtained samples were carried out by a Micromeritics ASAP 2460 analyzer. Atomic force microscopy (AFM) image was investigated by Bruker Dimension ICON with Nanoscope V controller. To measure the EM parameters, resultant products were mixed with PVDF in different mass percentages to be pressed into circular rings (Φ_out_ = 7.00 mm and Φ_in_ = 3.04 mm). Subsequently, it was tested with coaxial line method by a vector network analyzer (VNA; Agilent TE5071C).

## Results and Discussion

### Morphological Structures and Phase Crystallinity of LDHT and TMOT

**Fig. 1:**
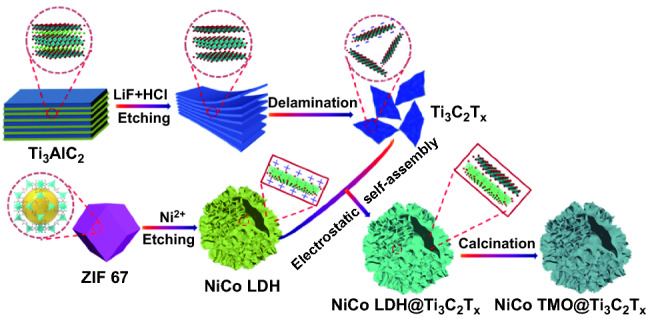
Schematic diagram of synthetic process for LDHT and TMOT

The preparation procedure and proposed structure of the novel 3D hollow LDHT and TMOT are shown in Fig. 1. The LDHT nanocages have been synthesized by utilizing ZIF-67 precursor as template and subsequent electrostatically self-assemble process. To obtain bimetallic NiCo LDH, the ZIF-67 templates are gradually etched by the H^+^ protons, meanwhile the Co^2+^ enable to be partially oxidized into Co^3+^ by the NO_3_^−^ and dissolved oxygen. Finally, the co-precipitation of both Co^2+^/Co^3+^ and Ni^2+^ facilitates the formation of the hollow NiCo LDH nanocages [29, 30]. Moreover, the LDHT nanocages can be transformed into TMOT nanocages after calcination treatment.

**Fig. 2:**
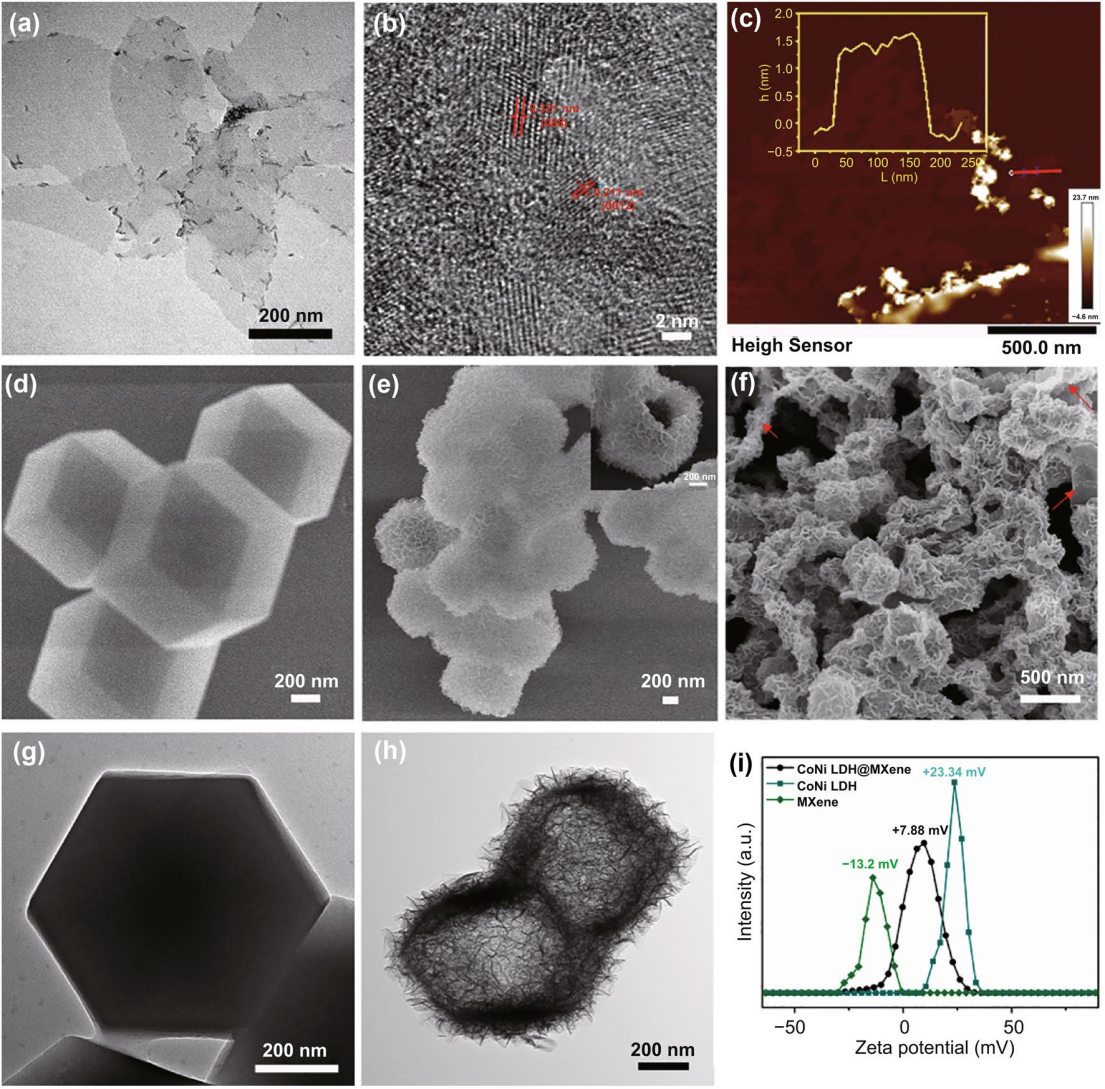
**a** TEM, **b** HRTEM, **c** AFM images of Ti_3_C_2_T_*x*_ nanosheets. **d** SEM, **g** TEM images of ZIF-67 precursors. **e** SEM, **h** TEM images of NiCo LDH nanocage. **f** SEM image of hollow LDHT nanostructure. **i** Zeta potentials image of Ti_3_C_2_T_*x*_ suspension, NiCo LDH and LDHT

Briefly, exfoliated Ti_3_C_2_T_*x*_ MXene nanosheets are successfully achieved by selectively etching the Al layers of the pristine bulk Ti_3_AlC_2_. The as-obtained Ti_3_C_2_T_*x*_ flakes have lateral size of 0.4–2.9 µm and thickness of 1.6 nm (Figs. 2a, c and S1a). High-resolution TEM (HRTEM, Fig. 2b) image reveals the distinct lattice fringes of 0.321 and 0.211 nm, corresponding to the (0008) and (0012) plane of Ti_3_C_2_T_*x*_ MXene. As shown in Fig. 2d, g, ZIF-67 precursors exhibit a rhombic dodecahedral structure with smooth surface and relatively uniform size of 700–800 nm. The average size of NiCo LDH is estimated approximately to be 850 nm, inheriting the polyhedral skeleton structure of ZIF 67, whose surface is constructed by a large number of ultrathin nanosheets (Fig. 2e). In addition, the enlarged SEM image (inset of Fig. 2e) and TEM image (Fig. 2h) clearly illustrate that the NiCo LDH particles display an inner hollow structure. Such porous and hollow nanocages composed of interconnected nanosheets endow large quantities of interfaces and heterojunctions, which are favorable to polarization and scattering of EM wave [31]. As shown in Fig. 2i, the Ti_3_C_2_T_*x*_ nanosheets are negatively charged with a zeta potential of − 13.2 mV derived from abundant surface functional groups (–O, –OH, and –F) caused by etching treatment, which endow MXenes with the capability to electrostatically decorate on NiCo LDH (+ 23.34 mV). Interestingly, large flocculation appears and settles down to the bottom of the container as introducing hollow NiCo LDH nanocages into MXene solution, indicating the electrostatic self-assembly between NiCo LDH and MXene. The as-synthesized LDHT composites with a Zeta potential of + 7.88 mV (Fig. 2i) emerge more clearly defined wrinkles on the surface and are connected to each other by thin MXene flakes marked with red arrows (Fig. 2f).

**Fig. 3:**
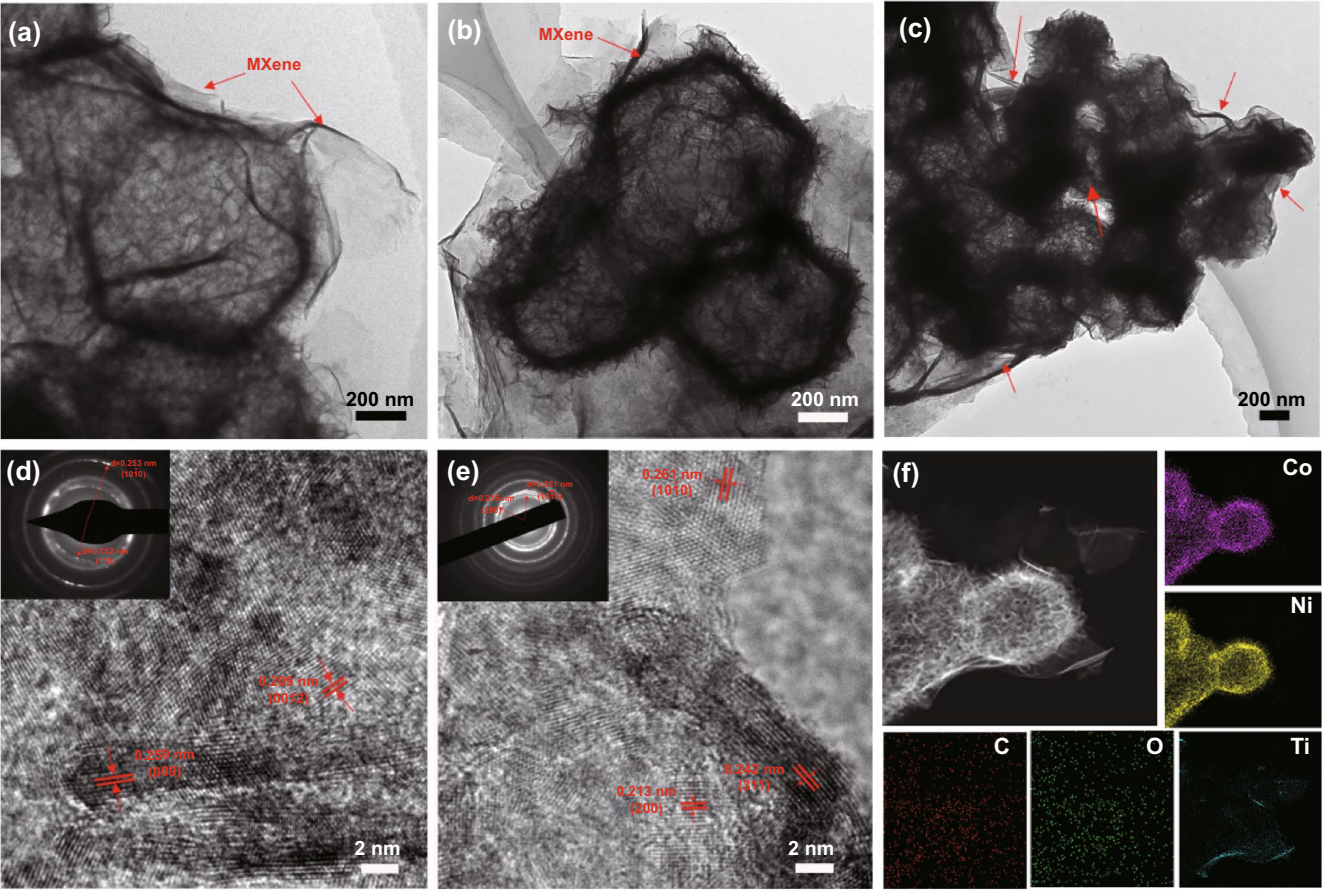
**a** TEM and **d** HRTEM images of LDHT. **b, c** TEM **e** HRTEM, and **f** element mapping images of TMOT

**Fig. 4:**
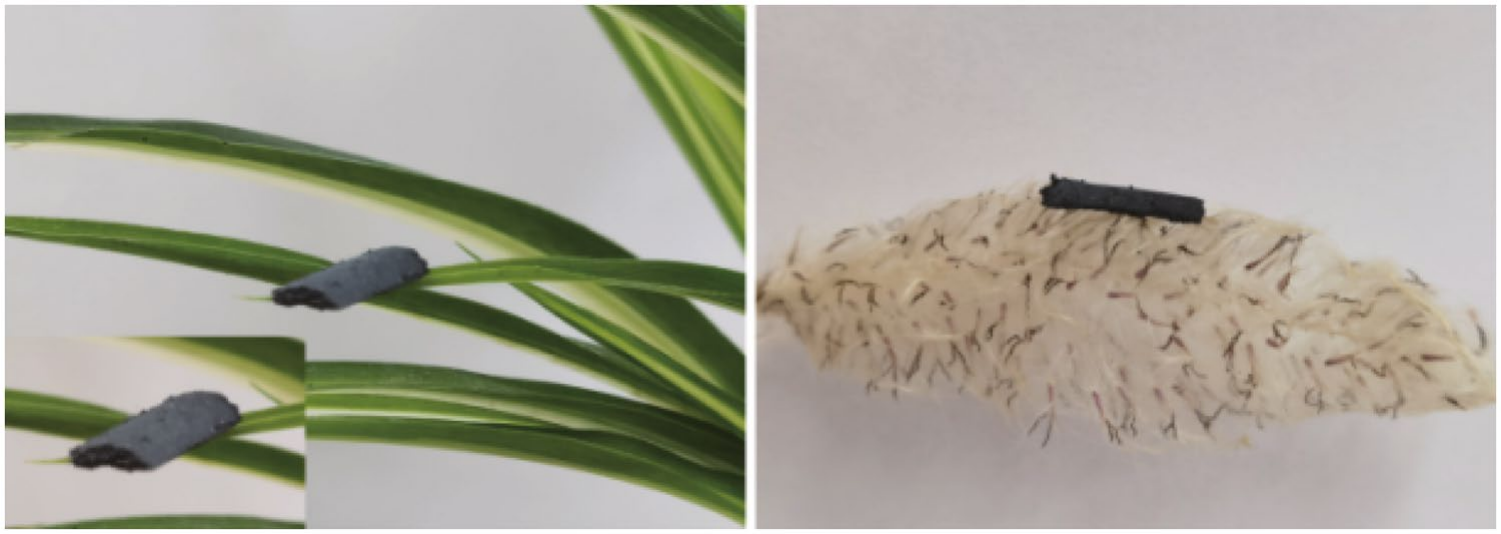
Images of LDHT composite on **a** leaf and **b** reed flocculants

This hollow hierarchical architecture is further illustrated by TEM and HRTEM. As shown in Fig. 3, the smaller Ti_3_C_2_T_*x*_ MXene nanosheets modify the surface of the hollow LDH/TMO structure, while the larger MXene nanosheets act as a connecting bridge, which is beneficial to the construction of the conductive network (Fig. 3a–c). In addition, TMOT products basically maintain the hollow polyhedron structure yet with slight collapse after calcination treatment (Fig. 3b). Two kinds of pronounced lattice fringes with lattice spacing of 0.209 nm and 0.259 nm are shown in Fig. 3d, corresponding to (0012) and (009) plane of Ti_3_C_2_T_*x*_ and NiCo LDH, respectively [32]. The selected area electron diffraction (SAED) pattern (inset in Fig. 3d) exhibits several rings, confirming the poly-crystalline structure of LDHT composites. For TMOT samples (Fig. 3e), lattice fringes of 0.261, 0.242, and 0.213 nm can be ascribed to the (10 $\overline{1}0$ ), (311), and (200) plane of Ti_3_C_2_T_*x*_, NiCo_2_O_4_, and CoO, respectively [33–35]. Moreover, the well-defined rings shown in the SAED image manifest the same results. The HRTEM images and the corresponding SAED images show LDHT/TMOT with good crystallinity. Besides, the element mapping analysis shown in Fig. 3f further demonstrates the uniform distribution of Co, Ni, Ti, C, and O, which reveal the MXene successfully assemble on the surface of TMO. Besides, the obtained LDHT flocculation with the bulk density of 0.0484 g cm^−3^ can stand on a leaf well, proving it is an ultralight material (Fig. 4).

**Fig. 5:**
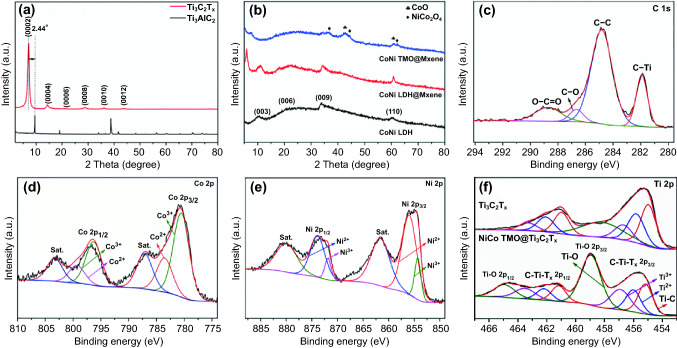
**a**, **b** XRD patterns of as-prepared samples, **c** C 1s, **d** Co 2p, **e** Ni 2p, and **f** Ti 2p spectra of TMOT and Ti_3_C_2_T_*x*_

The composition and structural characteristics of the as-obtained samples are identified by XRD. As shown in Fig. 5a, the disappearance of the most intense diffraction peak at about 39° in the Ti_3_C_2_T_*x*_ MXene pattern indicates that Al layers are selectively removed. Ti_3_C_2_T_*x*_ nanosheets display six peaks at 7.06°, 14.33°, 21.21° 28.75°, 36.12°, and 43.90°, which are assigned to the (0002), (0004), (0006), (0008), (0010), and (0012) crystal planes, respectively [36]. Moreover, the (0002) diffraction peak shifts to a lower angle in comparison with that of Ti_3_AlC_2_, proving the enlarged interlayer distance of the exfoliated Ti_3_C_2_T_*x*_ nanosheets [37]. The XRD pattern of ZIF 67 (Fig. S2) matches well with the previous pattern [38]. For NiCo LDH sample shown in Fig. 5b, the main peaks at 10.37°, 23.13°, 33.67°, and 59.90° are corresponding to the (003), (006), (009), and (110) planes of the typical LDH [39]. After electrostatic self-assembly, the new peak at 5.84° corresponding to (0002) represents the successful combination of NiCo LDH and Ti_3_C_2_T_*x*_ MXene nanosheets. The further increased interlayer spacing strongly suggests that the 3D hollow structure of the NiCo LDH efficiently alleviates the self-restacking of MXene nanosheets, which in turn enriches interfaces and prolongs the propagation path of EM wave. As for TMOT compounds (Fig. 5b), the diffraction peaks at 36.8° and 44.5° belong to the (311) and (400) planes of NiCo_2_O_4_ spinel phase (PDF No. 20-0781), respectively [30, 40]. Meanwhile, the diffraction peaks of 42.7° and 60.8° are associated with the (200) and (220) planes of CoO (PDF No. 71-1178), respectively.

The XPS is also conducted to prove the elemental composition and valence states information. It reveals that TMOT composites mainly comprise C, Ti, O, F, Co, and Ni elements in the survey spectrum (Fig. S4a), confirming the combination between NiCo TMO and Ti_3_C_2_T_*x*_ MXene nanosheets. Figure 5c exhibits that the binding energy of C 1 s at 288.71, 286.64, 284.82, and 281.92 could be assigned to O–C=O, C–O, C–C, and C–Ti of TMOT composites, respectively. The deconvolution of Co 2p spectrum (Fig. 5d) comprises two spin dipole orbitals (named Co 2p_3/2_, Co 2p_1/2_) and two shakeup satellites. The peaks at binding energy of 783.59 and 799.38 eV belong to Co^2+^ 2p_3/2_ and 2p_1/2_, while the peaks stood for Co^3+^ 2p_3/2_ and 2p_1/2_ present at 780.54 and 796.33 eV [41]. Analogously, the Ni 2p spectrum for TMOT composites (Fig. 5e) also exhibits two spin–orbit doublets. And the fitting peaks at approximately 873.85, 855.9, 871.82, and 854.31 eV are in agreement with Ni^2+^ 2p_1/2_, Ni^2+^ 2p_3/2_, Ni^3+^ 2p_1/2_, and Ni^3+^ 2p_3/2_, apart from the noticeable satellite peaks at 861.60 and 879.96 eV [42]. As shown in Fig. 5f, the Ti 2p relates four peaks of TMOT composites at 455.1, 455.9, 456.9, and 459.1 eV corresponding to Ti–C, Ti^2+^, Ti^3+^, and Ti–O, respectively [43]. The percentage of Ti–O bond increases from 4.28% in the Ti_3_C_2_T_*x*_ MXene to 41.54% in the TMOT, whereas no diffraction peak can be assigned to TiO_2_ in XRD pattern. It implies that the successful combination of Ti_3_C_2_T_*x*_ MXene and NiCo TMO is formed through strong covalent bond interaction [44]. Compared to the LDHT sample (Fig. S4f), the C–Ti–T_*x*_ content of TMOT composites decreases due to disappearance of surface functional groups during annealing process.

The nitrogen adsorption–desorption isotherms are generally employed to characterize the Brunauer–Emmett–Teller (BET) surface area and pore structures of as-obtained samples. The isotherm of all samples (Fig. S5) could be categorized as type IV with a distinct H3 hysteresis loop, which is considered to be a slit hole formed by the accumulation of lamellar particles. It is consistent with the SEM and TEM images of the sample. Furthermore, the pore size distribution of LDH, LDHT and TMOT composites is mainly at ≈ 2 nm. Plenty of pores can not only optimize the impedance matching, but also increase the flake-air interface, resulting in strong space charge polarization. The BET specific surface area of LDHT and TMOT composites calculated to be 83.94 and 68.47 m^2^ g^−1^ are larger than that of Ti_3_C_2_T_*x*_ (20.36 m^2^ g^−1^), which may effectively trap microwaves and aggregate charges in abundant interfaces leading to interfacial polarization as well as related relaxation.

### Microwave Absorbing Property of LDHT and TMOT

To realize the EM wave absorption properties of the LDHT and TMOT composites to the utmost extent, PVDF with unique dielectric property was chosen as the matrix. A dipole will be generated in the PVDF molecular chain derived from the different electronegativity of the H atom and the F atom, which will generate an electric dipole moment as well as dipole polarization under the action of an applied electric field [45]. As well, it is beneficial to construct flexible absorbers due to the inherent flexibility and wear resistance of PVDF (Fig. S6). As known, the MA property is strongly determined by its EM parameters, the complex relative permittivity (*ɛ*_*r*_, *ε*_*r*_ = *ε*′ − *jε*″) and permeability (*μ*_*r*_, *μ*_*r*_ = *μ*′ − *jμ*″) [46]. Hence, the electromagnetic parameters of various samples with the PVDF matrix were measured over the range of 2–18 GHz, and the data are demonstrated in Figs. S7 and S8. For the Ti_3_C_2_T_*x*_ sample, the values of *ε′* and *ε′′* are almost higher than those of the other samples over the whole frequency range. Obviously, with the increase in compound LDH content and decrease in filler loading (LDHT-x) in PVDF, both *ε′* and *ε′′* values are significantly reduced and deliver a similar declining trend with increasing frequency (Fig. S7). It manifests that the hyperactive conductivity of pure Ti_3_C_2_T_*x*_ MXene can be balanced by coupling with hollow LDH nanocages, which also optimizes the impedance matching and ultimately influences MA performance. One can see that two peaks appeared in the *ε″-f* curves of Ti_3_C_2_T_*x*_ and LDHT-x composites, indicating multiple resonant properties (Fig. S7b, e). A heterojunction capacitor will form in the interface between LDH and Ti_3_C_2_T_*x*_ MXene, which is conducive to the formation of resonance peak at ∼15 GHz and generate more interfacial polarization [17]. Meanwhile, the dielectric loss tangent (tan*δ*_*ε*_ = *ε*′′/*ε*′) and magnetic loss tangent (tan*δ*_*μ*_ = *μ*′′/*μ*′) related with the ability to convert EM energy were calculated to evaluate the microwave attenuation properties. The tan*δ*_*ε*_ values of LDHT-x and Ti_3_C_2_T_*x*_ MXene exhibit similar trends with the *ε*′′ and remain higher value about 15 GHz (Fig. S7c, f). Compared to changing LDHT-18 content in the PVDF, regulating the adherent Ti_3_C_2_T_*x*_ attachment can adjust the *ɛ*_*r*_ and tan*δ*_*ε*_ value in extremely slight way. By increasing the LDHT-18 content, massive interfaces including LDHT-18/PVDF, LDH/Ti_3_C_2_T_*x*_ and LDHT-18/LDHT-18 can serve as micro-capacitors with large capacity to store electric charge and enable high permittivity. In view of the non-magnetic property of the prepared material, the values of *μ*′ and *μ*″ (Fig. S8) are almost constant and close to 1 and 0, respectively. Importantly, LDHT-x composites possess higher dielectric loss values than magnetic loss, illustrating the dielectric loss plays a predominant role in the microwave attenuation performance.

Notably, dielectric loss inherently depends on conduction loss, multi-scattering and polarization relaxation that mainly arises from interface and dipole polarization [47, 48]. Ordinarily, the Debye dipolar relaxation process is conducted to reveal the mechanism of dielectric loss. According to Debye relaxation theory, the relationship between *ε*' and *ε*'' can be expressed as [3]:

1 $$\left( {\varepsilon^{\prime} - \frac{{\varepsilon_{s} + \varepsilon_{\infty } }}{2}} \right)^{2} + \left( {\varepsilon^{\prime\prime}} \right)^{2} = \left( {\frac{{\varepsilon_{s} - \varepsilon_{\infty } }}{2}} \right)^{2}$$

where *ε*_*s*_ and *ε*_∞_ represent static dielectric permittivity and the relative permittivity at an infinite frequency, respectively. It can be concluded that the *ε*' and *ε*'' curves can form a semicircle marked as Cole–Cole semicircle, and each semicircle reveals a Debye relaxation process. Obviously, multiple twisted Cole–Cole semicircles are observed in 2–18 GHz from hollow LDHT-x/PVDF composites (Fig. S9c–f), authenticating the multiple polarization relaxation behaviors. These relaxation processes include the dipole polarization of surface groups on Ti_3_C_2_T_*x*_, bonding charge and interface polarization induced by abundant heterogeneous interfaces and the porous hollow structure.

Reflection loss (RL) is calculated to evaluate intuitively the EM wave absorption abilities, which is in line with the transmission line theory. The formula is described as eq. [49]:

2 $${\text{RL}} = 20\lg \left| {\frac{{Z_{{{\text{in}}}} - Z_{0} }}{{Z_{{{\text{in}}}} + Z_{0} }}} \right|$$

3 $$Z_{{{\text{in}}}} = Z_{0} \sqrt {\frac{{\mu_{r} }}{{\varepsilon_{r} }}\tanh \left( {j\frac{2\pi fd}{c}\sqrt {\mu_{r} \varepsilon_{r} } } \right)}$$

where *Z*_in_ and *Z*_0_ are is the input impedance of the absorber and free space, respectively. *f* is the frequency of EM wave, *c* is the light velocity in the free space, and *d* is the thickness of the absorbing layers, respectively.

**Fig. 6:**
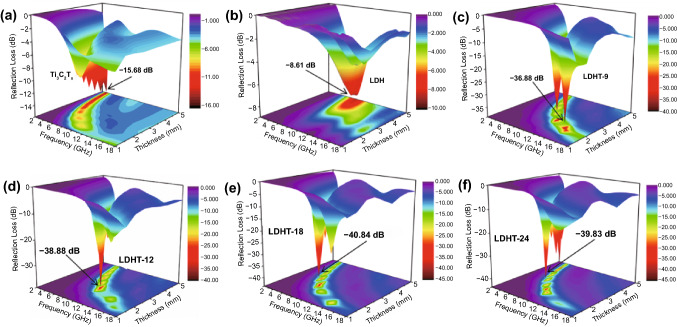
3D RL values of **a** Ti_3_C_2_T_*x*_, **b** LDH, **c** LDHT-9, **d** LDHT-12, **e** LDHT-18 and **f** LDHT-24 composites with a filler content of 15 wt% at different thicknesses

**Fig. 7:**
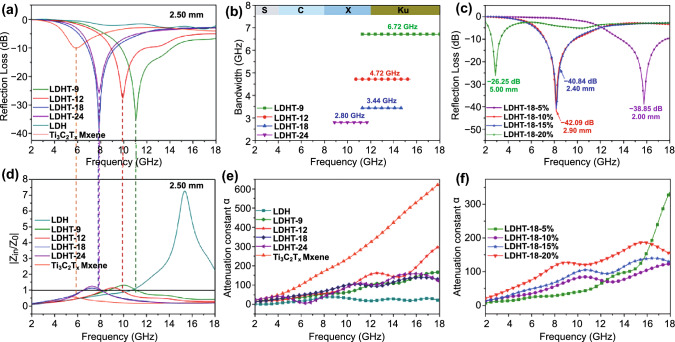
**a** 2D curves of RL at 2.50 mm, **b** EAB with the filler content of 15 wt%. **c** RL curves at different loading in PVDF. **d** characteristic impedance curves, **e** attenuation constant of as-prepared samples in 15 wt%. **f** attenuation constant at different loading in PVDF

As for the laminar Ti_3_C_2_T_*x*_ MXene shown in Fig. 6a, it merely exhibits negligible microwave absorption capacity with a minimum reflection loss value of − 15.68 dB, which is originated from the incompatibility between high conductivity and impedance matching. Similarly, unsatisfied RL_min_ value of − 8.61 dB is also emerged in porous NiCo LDH with hollow structure (Fig. 6b). On account of the effective synergies among hollow hierarchical architectures as well as multi-components of Ti_3_C_2_T_*x*_ MXene and porous NiCo LDH nanoparticles, LDHT-x realizes better MA properties than that of Ti_3_C_2_T_*x*_ MXene and LDH (Fig. 6c–f). Particularly, LDHT-9 absorber (Fig. 6c) displays the RL_min_ value of − 36.88 dB and remarkably achieves an unexpected effective absorption bandwidth of 6.72 GHz at only 2.10 mm, covering the entire Ku-band. Specifically, LDHT-18 absorber represents the outstanding MA ability with RL_min_ value of − 40.84 dB and wide EAB of 3.28 GHz covering almost 82% of the X-band (Figs. 6e and S11). It is clearly observed that the filler loading of LDHT-x in PVDF has distinctly regulative effect on the MA performance (Fig. S10). Besides, the optimal filler content is 15 wt%. In conclude, adjusting the attachment Ti_3_C_2_T_*x*_ flakes can subtly regulate the absorbing property with tune, the lowest RL_min_ and their corresponding frequency band. This phenomenon is consistent with the variation of electromagnetic parameters. Furthermore, the RL_min_ shifts to lower frequency accompanied by a reduction in EAB as increasing Ti_3_C_2_T_*x*_ nanoflakes decoration at the same thickness of 2.5 mm with the filling content of 15 wt% (Fig. 7a, b). Obviously, the optimal RL_min_ value of LDHT-18 also follows this pattern (Fig. 7c), and this reminds us that changing filler content and Ti_3_C_2_T_*x*_ MXene attachment are available to obtain a tunable and high-efficient microwave absorption material with broaden EAB and thin thickness.

To clearly clarify the microwave absorption enhancement mechanism of LDHT-x/PVDF composites, the normalized characteristic impedance (*Z* =|*Z*_in_/*Z*_0_|), which determines whether the electromagnetic wave can enter the material, and attenuation constant (*α*) related to comprehensive loss competence of absorbers are shown in Fig. 7d–f. Among them, the attenuation constant (*α*) can be figured out by equation [50]:

4 $$\alpha = \frac{\sqrt 2 \pi f}{c}\sqrt {\left( {\mu^{\prime\prime}\varepsilon^{\prime\prime} - \mu^{\prime}\varepsilon^{\prime}} \right) + \sqrt {\left( {\mu^{\prime\prime}\varepsilon^{\prime\prime} - \mu ^{\prime}\varepsilon ^{\prime}} \right)^{2} + \left( {\mu^{\prime}\varepsilon^{\prime\prime} + \mu ^{\prime\prime}\varepsilon ^{\prime}} \right)^{2} } }$$

As known, the impedance matching is the primary consideration for the designs of excellent EM wave absorber [51]. Generally, the impedance matching is greatly affected by the complex permittivity and the complex permeability [52, 53]. Thus, reasonable regulation of EM parameter through adjusting the content of compound LDH and filler loading of LDHT-x in PVDF could be effective in achieving impedance matching. As shown in Fig. 7d, the *Z* results reveal that the LDHT-x with loading of 15 wt% in PVDF, especially for LDHT-18 composites, obtain the improved impedance matching with more regions closing to or equaling 1 compared with individual LDH and Ti_3_C_2_T_*x*_ MXene. In this case, LDHT-x will allow as much radiated EM wave as possible to enter its interior, paving the way for the subsequent consumption and absorption. Meanwhile, the peak of Z values shifts to the lower frequency as increasing decorated of Ti_3_C_2_T_*x*_ MXene, which are consistent with the RL_min_ in Fig. 6a. The attenuation constant (*α*) is another key factor to determine the EM absorbing performance as shown in Fig. 7e, f. The LDH sample displays the lowest *α* value, which reveals the weakest attenuation ability to incident microwaves. And the α values of LDHT-x composited are significantly enhanced after combining with Ti_3_C_2_T_*x*_ MXene, and show a slight fluctuation as regulating Ti_3_C_2_T_*x*_ MXene loading. Figure 7f further illustrates the effects of the different filler loadings on attenuation ability. The values of *α* are distinctly improved with increasing LDHT-18 loading from 5 to 20 wt%. This is also in agreement with the results of EM parameters and refection loss.

**Fig. 8:**
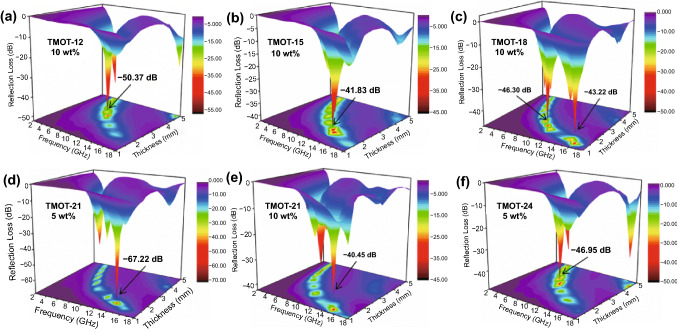
3D plots of RL values for various TMOT-x composites

In accordance with the above findings, the EM parameter and MA ability of LDHT-x can be slightly adjusted by regulating the adherent Ti_3_C_2_T_*x*_ nanoflakes amount. Therefore, TMOT-x hollow architectures with more compound proportions were prepared by pyrolyzing the corresponding LDHT-x composites to further improve the microwave attenuation capacity. It is obvious that TMOT-x absorbers possess remarkably enhanced EM absorbing properties with lower RL_min_ and filler content in PVDF compared with the LDHT composite (Figs. 8 and S12). Besides, the MA performance of TMOT-x absorbers are not desirable when the filler loading increases to 15 wt%, which may be attributed to high-frequency oscillating skin current and reflection of incident microwave caused by excessive 3D conductive network [15, 54]. The absorption peak gradually moves to the low frequency and low filling amount with increasing the Ti_3_C_2_T_*x*_ MXene attachment. To be specific, as shown in Fig. 8a, TMOT-12 absorber could achieve excellent MA properties with the reflection loss of − 50.37 dB and the corresponding EAB of 3.44 GHz (7.12–10.56 GHz) at 3.20 mm. When the volume of Ti_3_C_2_T_*x*_ MXene suspension increases to 15 mL, the effective EM absorption occurs at lower filling mass fraction of 5 wt% (Fig. S12). And the optimal filler content is trended toward 5 wt% as increasing continuously the anchored Ti_3_C_2_T_*x*_ MXene flake. Furthermore, TMOT-18 absorber exhibit RL_min_ of − 43.22 and −  46.30 dB at 1.40 and 2.20 mm, respectively (Fig. 8c). Surprisingly, for TMOT-21 absorber as shown in Fig. 8d, it remarkably emerges an unexpected RL_min_ value of − 67.22 dB corresponding to the absorption bandwidth of 3.84 GHz at 1.70 mm. When the thickness changes to 4.00 mm, the RL_min_ value reaches − 48.37 dB at low frequency (5.6 GHz) within only 5 wt% filler loading. Thus, TMOT-21 absorber equips with absolute advantages on light weight and ultrathin thickness, and realizes splendid MA performance with remarkable RL_min_ and tunable EAB.

**Fig. 9:**
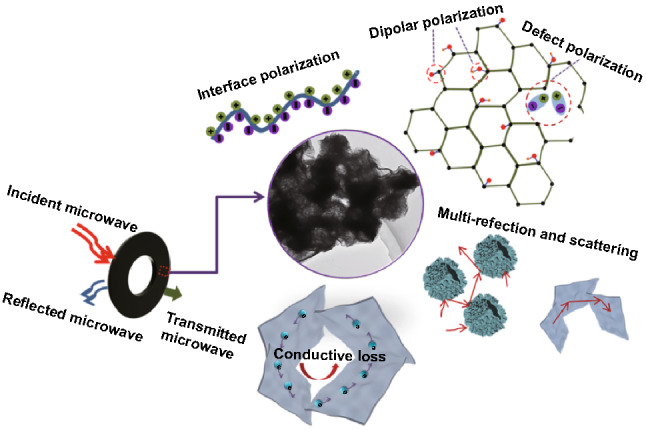
Schematic illustration of microwave absorption mechanism for TMOT

The underlying MA mechanisms of the unique 3D hollow hierarchical NiCo compound@MXene composites are shown in Fig. 9. The hollow layered structure with open void space will optimize impedance matching behavior and contribute to lightweight absorber as well as generate more interfacial polarization [55]. Besides, bending Ti_3_C_2_T_*x*_ MXene sheets with random orientation is beneficial to construct 3D conductive network to enable free migration of electrons in conductive pathways, which would further enhance the conduction loss to consume the EM wave by converting EM energy into thermal energy [56]. Simultaneously, 3D porous hierarchical NiCo compound@MXene composites can provide multiple reflection and scattering to prolong the transmission path of electromagnetic waves, leading to the dissipation of electromagnetic energy [43, 53]. Meanwhile, substantial termination functional groups and intrinsic defects of layered Ti_3_C_2_T_*x*_ MXene as well as the oxygen vacancies of NiCo TMO/LDH caused by calcination effectively optimize the impedance matching, while giving rise to the enhanced dipole polarization and defect polarization. The 3D hierarchical structure assembled by Ti_3_C_2_T_*x*_ MXene flakes and hollow NiCo TMO/LDH can offer high density of heterogeneous interfaces, which will induce accumulation and uneven distribution of space charges and produce a macroscopic electric moment leading to remarkable interfacial polarization (called as the Maxwell–Wagner effect) [57].

Finally, the optimized RL values (ORL_1_) are introduced to reasonably evaluate MA ability as considering filler loading [58]:

5 $${\text{ORL}}_{1} = \frac{{{\text{RL}}}}{{\text{filler loading}}}$$

**Fig. 10:**
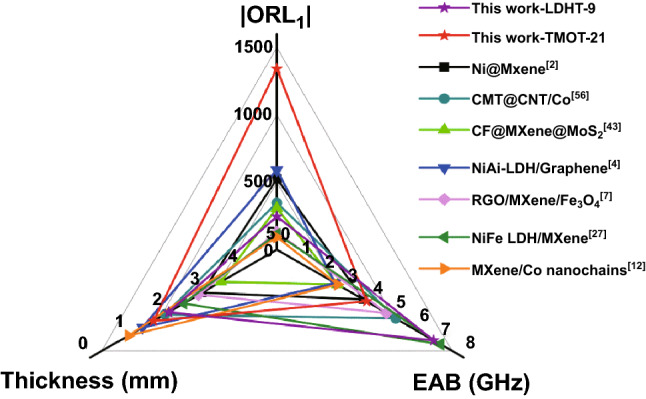
Comparison of the MA properties of various related materials

Compared with other works, a desirable effective absorption bandwidth of LDHT-9 absorber is up to 6.72 GHz with the thickness of 2.10 mm, which can be comparable to the progressive absorbing material (Fig. 10). It is worth noting that TMOT-21 absorber achieves inspiring MA performance with the RL_min_ value of − 67.22 dB and EAB of 3.84 GHz at 1.70 mm under extremely low filling content of 5 wt%. And the ORL_1_ value of TMOT-21 absorber almost surpasses various kinds of advanced absorbents reported by previous literatures, which is closer to the ideal absorbing material.

## Conclusion

In summary, the unique 3D NiCo compound@MXene that inherited the hollow polyhedral skeleton structure and excellent conductive network has been successfully synthesized by facile electrostatically assembling method and calcination treatment. The formation of hierarchical architecture efficiently prevents the restacking of 2D Ti_3_C_2_T_*x*_ nanosheets and further enlarges the specific surface area which is beneficial to the conduction loss and polarization. It is concluded that the EM parameters and MA performance can be regulated by the adherent Ti_3_C_2_T_*x*_ content and filler loading amount in PVDF matrix. The LDHT-9 remarkably possesses ultra-wide EAB of 6.72 GHz at the thickness of 2.10 mm. The TMOT-21 near the percolation threshold gives rise to brilliant EM absorbing ability with an optimal RL_min_ value of − 67.22 dB and EAB of 3.84 GHz at 1.70 mm with only 5 wt% loading. Besides, the combination of the hybrids and PVDF endow the absorber with flexibility and wear resistance as well. Thus, this work provides inspiration to construct 3D hollow hierarchical structure and efficiently moderate electromagnetic parameters, which might help for promising candidate in ultralight and ultrathin MA absorbers.

## Supplementary Information

Below is the link to the electronic supplementary material.

TEM, SEM, XRD, elemental mappings, XPS, BET, Optical image, Permittivity, Permeability, Cole−Cole curves, Reflection loss, Effective absorbing bandwidth, C0 and relative input impedance of this work. (PDF 2017 KB)
