# Supplementary material for: 3D Ultralight Hollow NiCo Compound@MXene Composites for Tunable and High-Efficient Microwave Absorption
Source: Nanomicro Lett. 2021 Oct 11;13:206. doi: 10.1007/s40820-021-00727-y (PMC8505608; doi:10.1007/s40820-021-00727-y)
Supplement: Supplementary file 1 — TEM, SEM, XRD, elemental mappings, XPS, BET, Optical image, Permittivity, Permeability, Cole−Cole curves, Reflection loss, Effective absorbing bandwidth, C0 and relative input impedance of this work. (PDF 2017 KB) [file 40820_2021_727_MOESM1_ESM.pdf]

Supporting Information for

3D Ultralight Hollow NiCo Compound@MXene Composites for Tunable and High-efficient Microwave Absorption

Hui-Ya Wang[1], Xiao-Bo Sun[1], Shu-Hao Yang[1], Pei-Yan Zhao[1], Xiao-Juan Zhang[2,]*, Guang-Sheng Wang[1,]*, Yi Huang[3]

[1] School of Chemistry, Beihang University, Beijing 100191, P. R. China

[2] College of Chemistry and Materials Engineering, Beijing Technology and Business University, Beijing 100048, P. R. China

[3] School of Materials Science and Engineering, Nankai University, Tianjin 300350, P. R. China

*Corresponding authors. E-mail: wanggsh@buaa.edu.cn (Guang-Sheng Wang); zhxiaojuan@btbu.edu.cn (Xiao-Juan Zhang)

Supplementary Tables and Figures

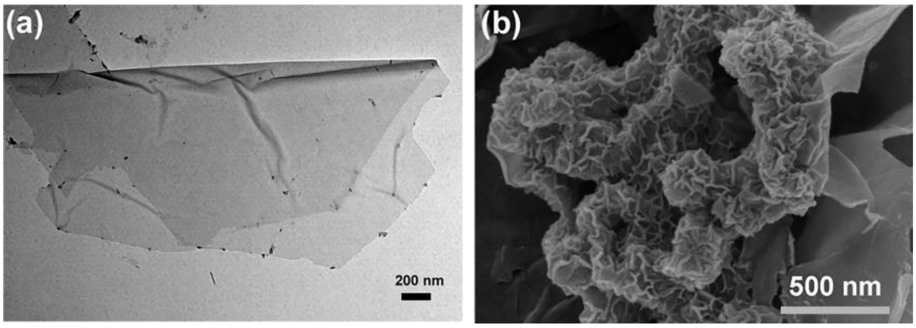

Fig. S1 a TEM images of $Ti_3C_2T_x$ nanosheets. **b** SEM image of LDHT composites

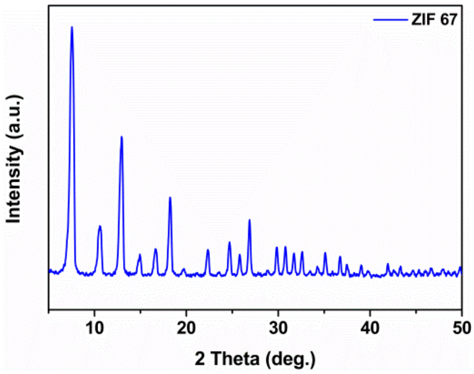

Fig. S2 XRD patterns of ZIF 67 precursor

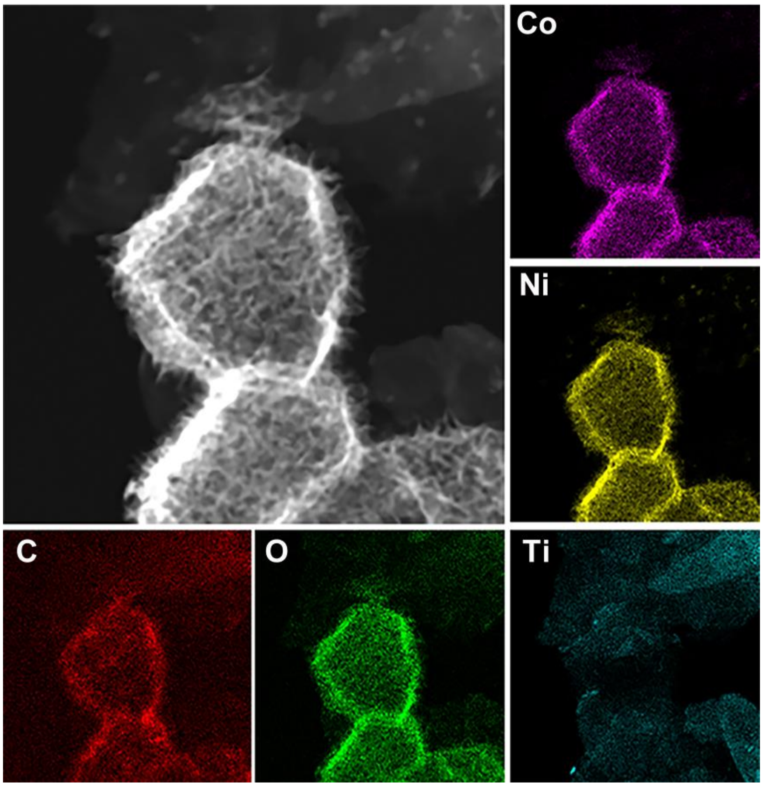

Fig. S3 Element mapping images of LDHT

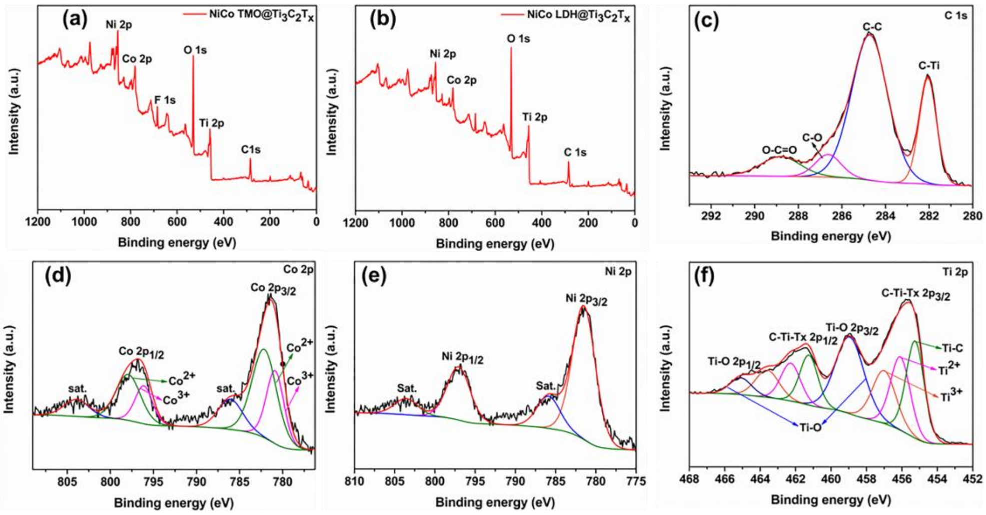

Fig. S4 a, b X-ray photoelectron spectroscopy (XPS) survey of TMOT and LDHT composites. **c** C 1s, **d** Co 2p, **e** Ni 2p, **f** Ti 2p XPS spectra of LDHT composites

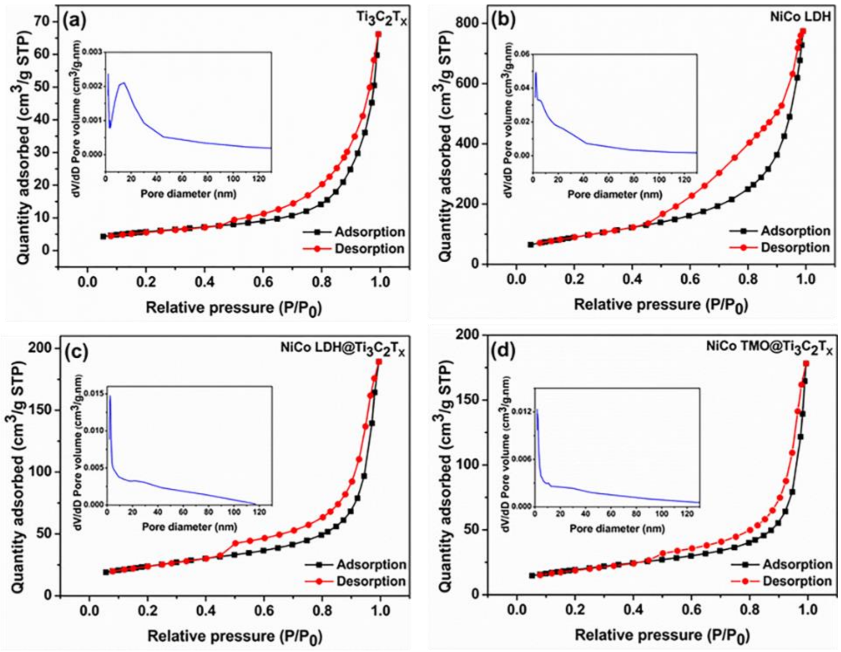

Fig. S5 Nitrogen adsorption–desorption isotherms and the pore diameter distribution: **a** $Ti_3C_2T_x$, **b** NiCo LDH, **c** LDHT and **d** TMOT composites

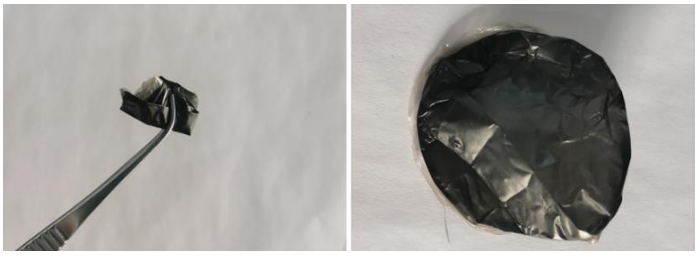

Fig. S6 Images of LDHT flexible film absorbers

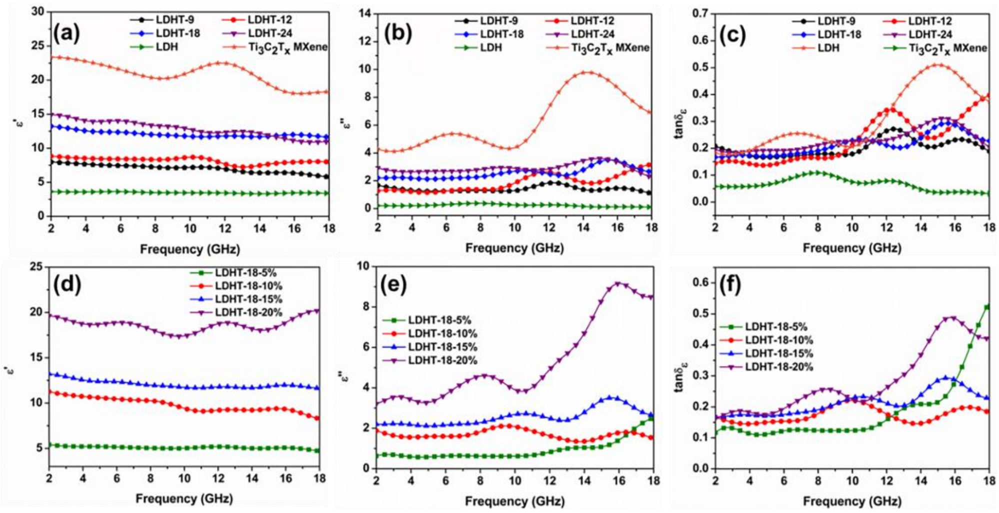

Fig. S7 Frequency dependence of complex permittivity for various composites: **a** Real part ε', **b** imaginary part ε'', **c** dielectric loss tangent of $Ti_3C_2T_x$, LDH and LDHT-x composites with filler content of 15 wt%. **d** Real part ε', **e** imaginary part ε'', **f** dielectric loss tangent of LDHT-18 composites at different loadings

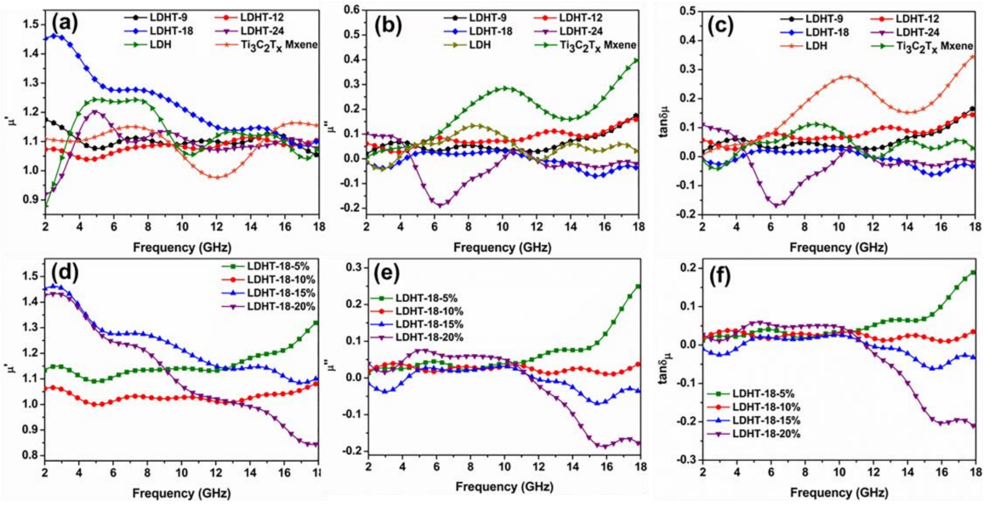

Fig. S8 **a** Real part μ', **b** imaginary part μ'', **c** magnetic loss tangent of complex permeability for $Ti_3C_2T_x$, LDH and LDHT-x composites with filler content of 15 wt% **d** real part μ', **e** imaginary part μ'', **f** magnetic loss tangent of complex permeability for LDHT-18 composites at different loadings

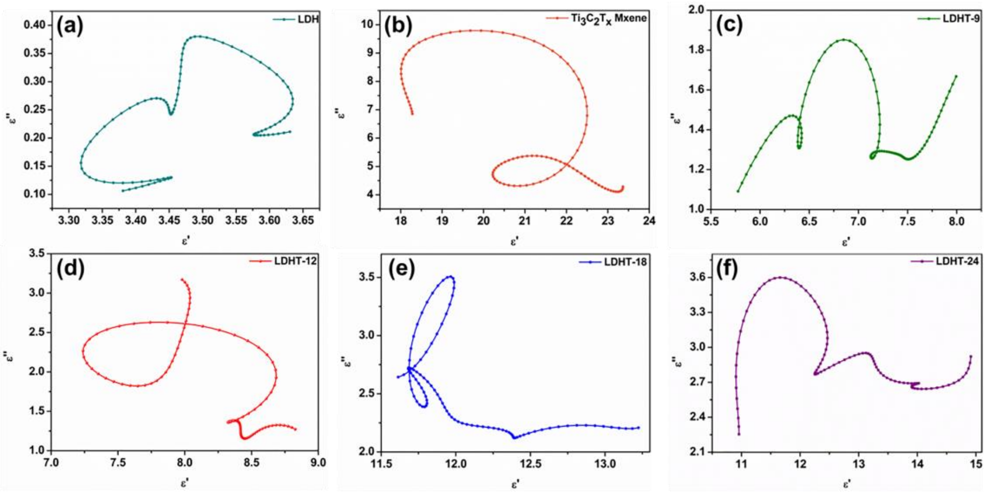

Fig. S9 Relationship between real and imaginary dielectric part of various samples: **a** LDH, **b** $Ti_3C_2T_x$, **c** LDHT-9, **d** LDHT-12, **e** LDHT-18, **f** LDHT-24

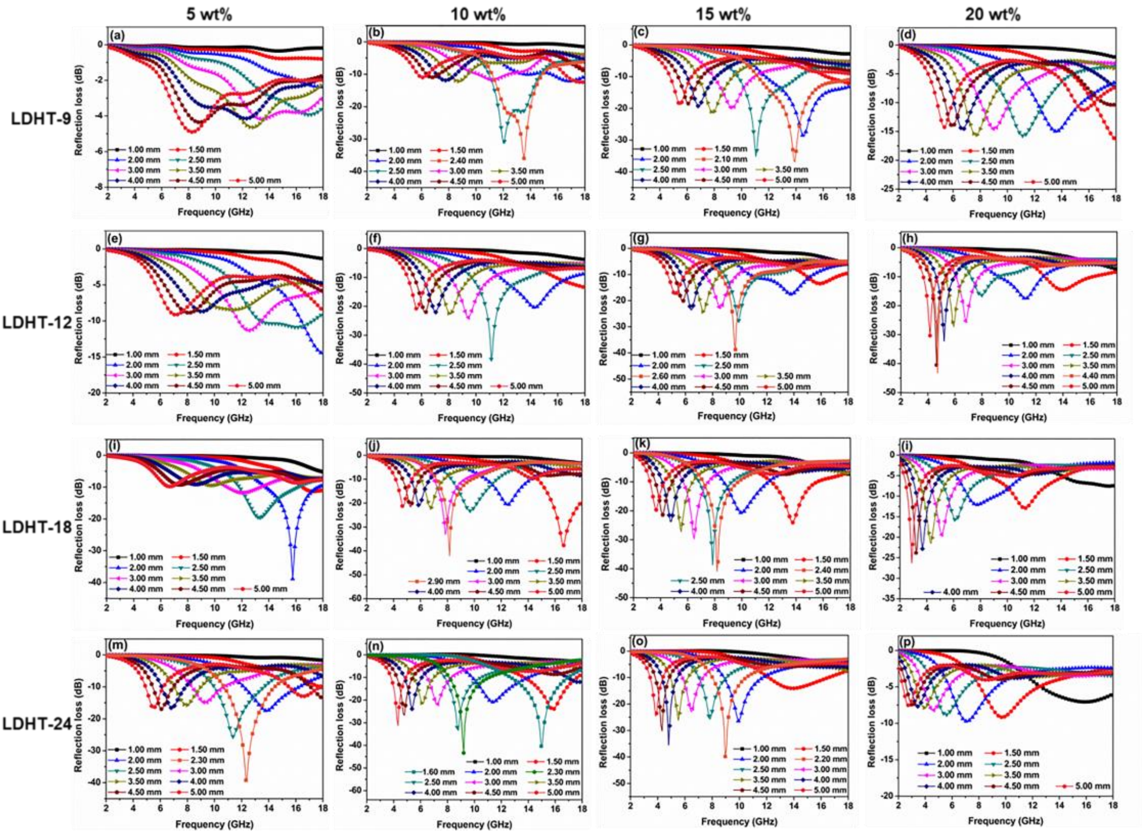

Fig. S10 2D RL curves of the LDHT-x series

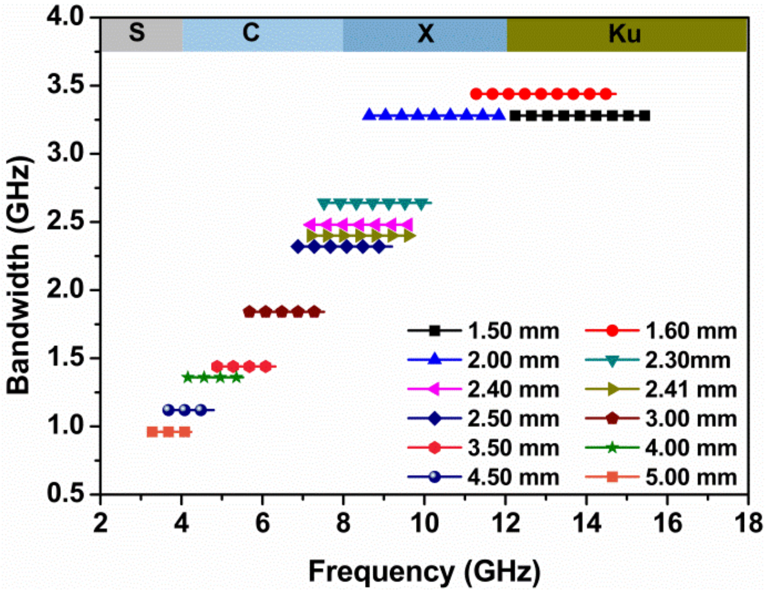

Fig. S11 Effective absorbing bandwidth of LDHT-18 at different thicknesses composites with 15 wt% filler content

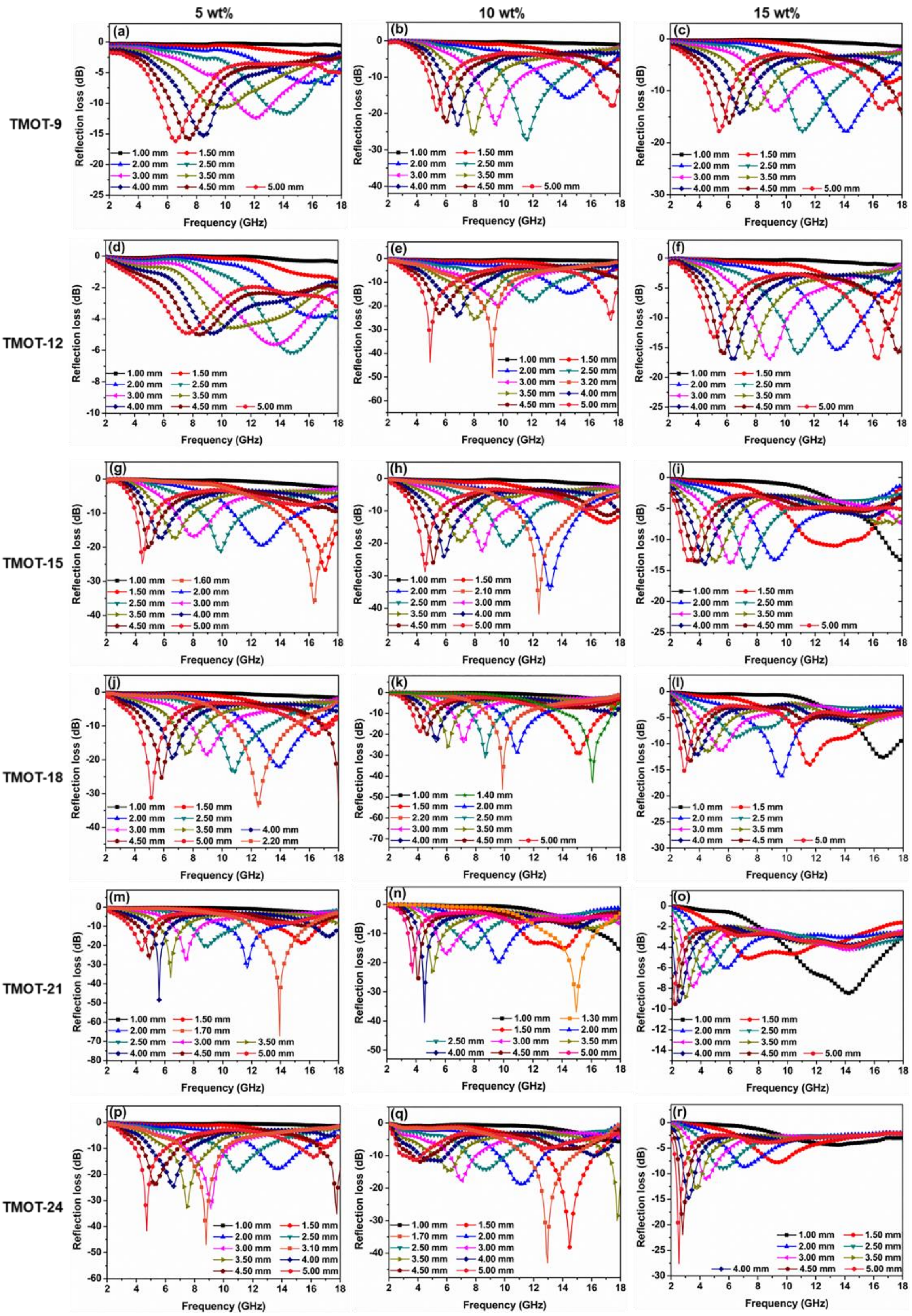

Fig. S12 2D RL curves of the TMOT-x series

Overall, the ε′ values of all TMOT-x samples exhibit a downward trend with the increasing frequency revealing a typical frequency dispersion phenomenon and are intimately determined to the load of $Ti_3C_2T_x$ (Fig. S13). With raised $Ti_3C_2T_x$ decoration, the ε' and ε'' values first increased and then decreased, which is attributed to increase in current leakage engendered from the increased area of MXene nanosheets contact [S1, S2]. By contrast, the dielectric properties of TMOT-x samples are enhanced than those of LDHT-x composites, mainly due to the large number oxygen vacancies which act as polarization center and are beneficial to electromagnetic wave absorption. During the calcination process, part of the Co^{3+} is reduced to Co^{2+} as proved in XPS spectra, resulting in disordered lattice arrangement and formation of oxygen vacancies. Interestingly, the presence of oxygen vacancies will bind the surrounding electrons and act as electron donors, facilitating charge transfer and separation to generate polarization center [S3]. As illustrated in Fig. S13d-f, the values of μ' and μ'' for all TMOT-x composites basically hold similar change trends and suddenly decrease over 2.0–7.0 GHz and then fluctuate in a gentle way, indicating that the magnetic loss also make a relevant contribution to microwave absorption. As represented in Fig. S13 c,f, it is no surprise that the variation trends of $\tan\delta_\varepsilon$ and $\tan\delta_\mu$ are similar with these of ε' and μ''.

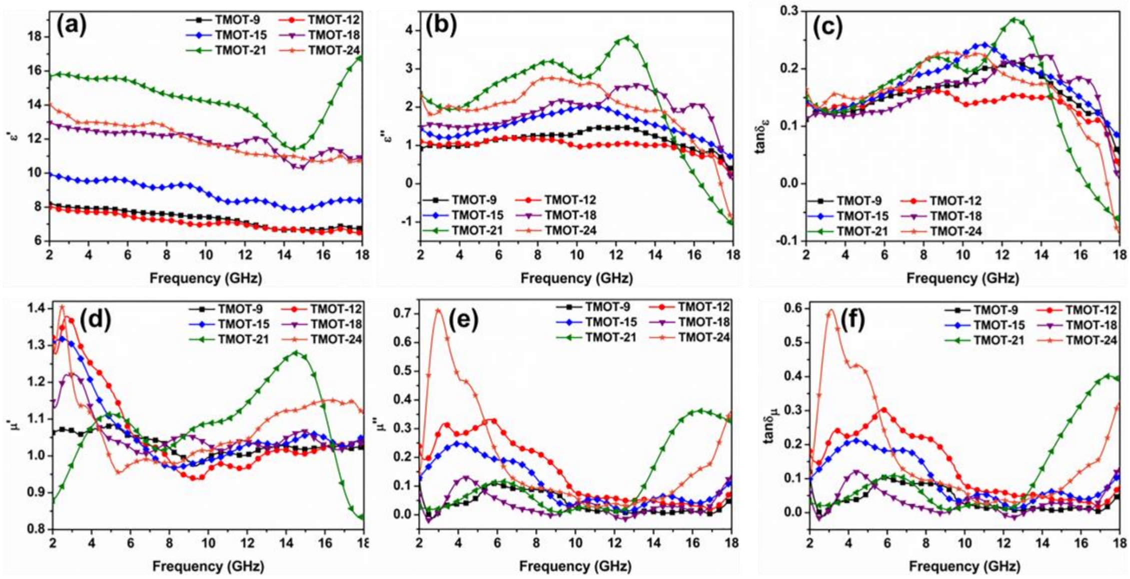

Fig. S13 a Real part ε′, **b** imaginary part ε′′, **c** dielectric loss tangent of complex permittivity, **d** real part μ', **e** imaginary part μ'', **f** magnetic loss tangent of complex permeability of TMOT-x composite with the filler loading of 10 wt%

Generally, the magnetic loss mainly comes from natural resonance, exchange resonance and eddy current loss in 2-18 GHz, whereas the domain wall resonance usually generated in the 1−100 MHz and the hysteresis loss in this weak magnetic field can be neglected. Universally, the eddy current loss can be evaluated by the values of the eddy current coefficient C_0($C_0=\mu''(\mu')^{-2}f^{-1}$) and shown in Fig. S15. If C_0 is a constant as the frequency changes, the eddy current loss should be the individual loss mechanism. However, it is clear that TMOT-x appears intensive fluctuations in the range of 2-10 GHz, elucidating that both the eddy current loss and natural resonance loss contributes

to the magnetic loss. The values of attenuation constant increase linearly with the increase of frequency, and improve slightly with the increase of $Ti_3C_2T_x$ adherent, excluding TMOT-24-10%. Besides, the α values of TMOT-x samples also enhance compared with LDHT-x composites at the same filler loading, which is beneficial to optimize microwave absorption property. For TMOT-21 composites with 5 wt% filler loading, the impedance matching effect occurs optimally at a thickness of 1.70 mm, meanwhile the RL_{min} predictably exceeded -67.22 GHz as shown in Fig. S16.

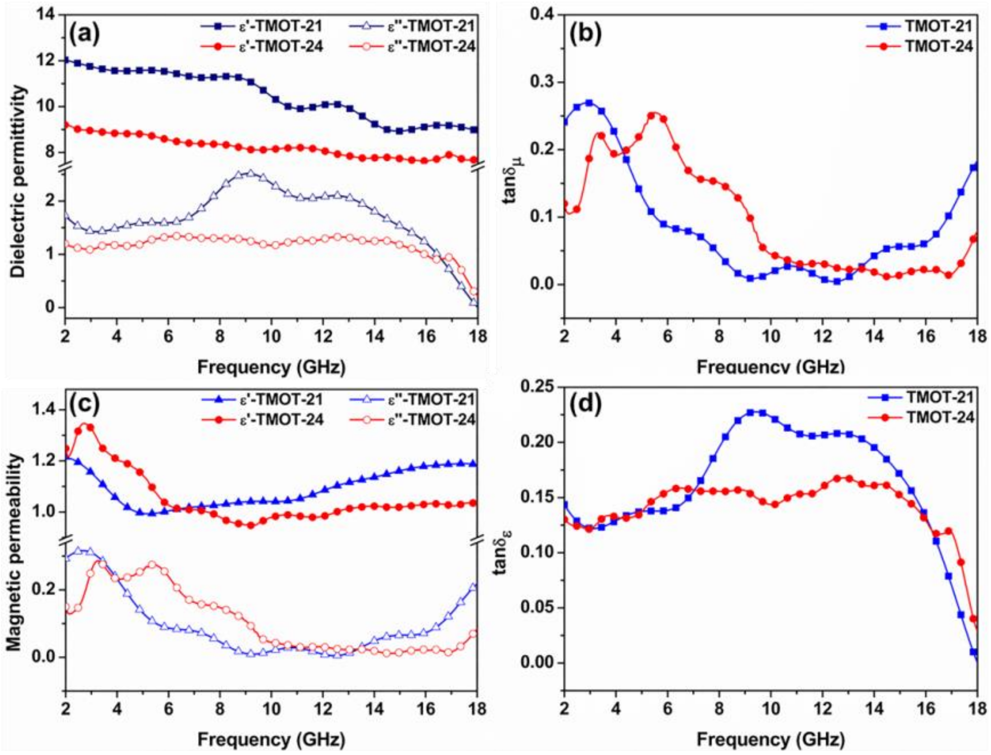

Fig. S14 Electromagnetic parameters of TMOT-21 and TMOT-24 composites with the filler loading of 5 wt%: **a** complex permittivity, **b** dielectric loss tangent, **c** complex permeability and **d** magnetic loss tangent of TMOT-x composites

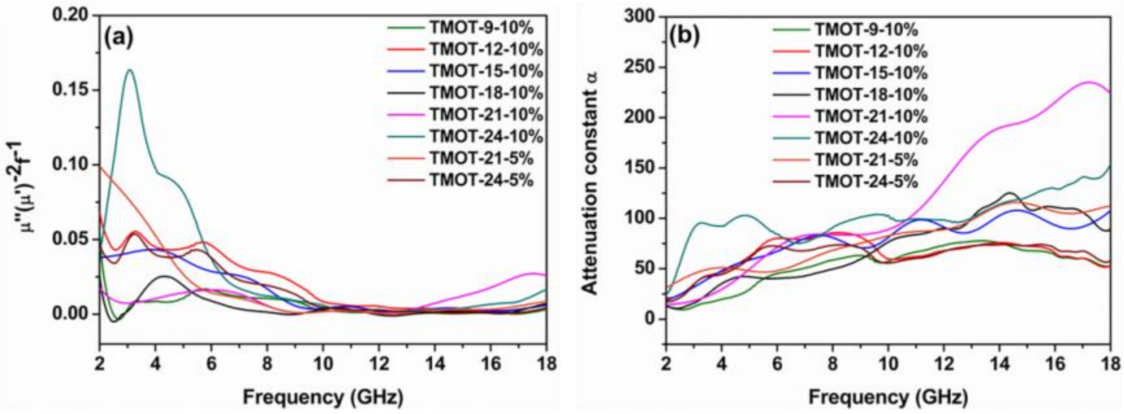

Fig. S15 a Calculated $\mu''(\mu')^{-2}f^{-1}$, **b** α value of series TMOT composites over the entire frequency of 2-18 GHz

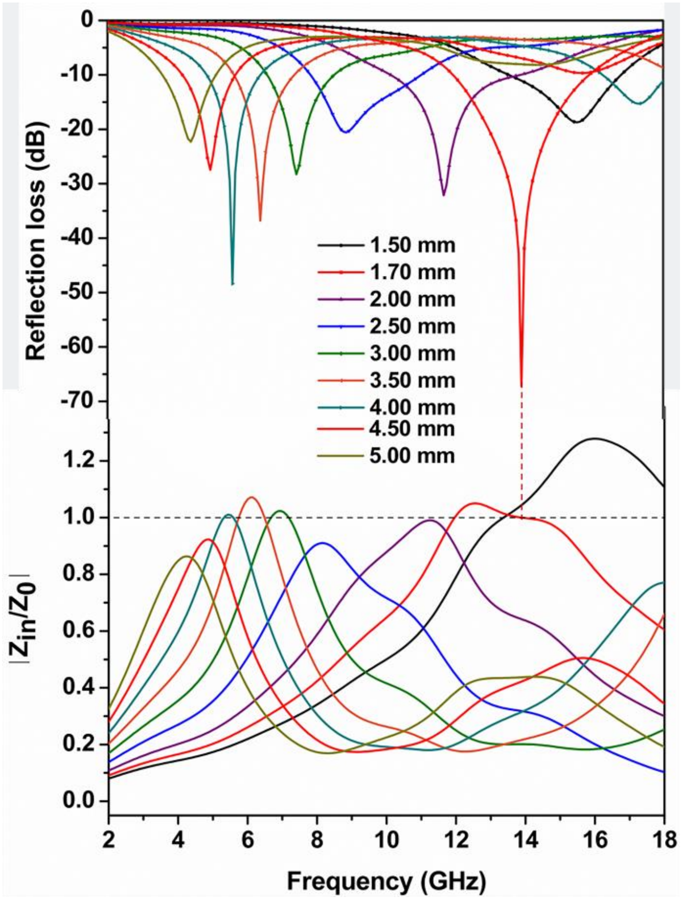

Fig. S16 Frequency dependence of RL and the relative input impedance ($|Z_{in}/Z_0|$) curves of TMOT-21 with 5 wt% filler loading

Supplementary References

[S1] Z. Wang, Z. Cheng, C.Q. Fang, X.L. Hou, L. Xie, Recent advances in MXenes composites for electromagnetic interference shielding and microwave absorption. Compos. Part A Appl. Sci. Manuf. **136**, 105956 (2020). https://doi.org/10.1016/j.compositesa.2020.105956

[S2] M.S. Cao, Y.Z. Cai, P. He, J.C. Shu, W.Q. Cao et al., 2D MXenes: Electromagnetic property for microwave absorption and electromagnetic interference shielding. Chem. Eng. J. **359**, 1265-1302 (2019). https://doi.org/10.1016/j.cej.2018.11.051

[S3] T.Q. Hou, B.B. Wang, M.L. Ma, A.L. Feng, Z.Y. Huang et al., Preparation of two-dimensional titanium carbide ($Ti_3C_2T_x$) and $NiCo_2O_4$ composites to achieve excellent microwave absorption properties. Compos. Part B Eng. **180**, 107577 (2020). https://doi.org/10.1016/j.compositesb.2019.107577
